# Fatal neonatal hypertrophic cardiomyopathy caused by compound heterozygous truncating *MYBPC3* mutation

**DOI:** 10.1007/s12471-019-1245-2

**Published:** 2019-02-11

**Authors:** S. Alsters, L. Wong, L. Peferoen, H. W. M. Niessen, H. Bikker, M. W. Elting, A. C. Houweling

**Affiliations:** 1 Department of Clinical Genetics, Amsterdam UMC, Vrije Universiteit Amsterdam, Amsterdam, The Netherlands; 2 Department of Cardiology, Amsterdam UMC, Vrije Universiteit Amsterdam, Amsterdam, The Netherlands; 3 Department of Pathology, Amsterdam UMC, Vrije Universiteit Amsterdam, Amsterdam, The Netherlands; 4 Department of Clinical Genetics, Amsterdam UMC, Academic Medical Center, Amsterdam, The Netherlands

We report the clinical and genetic findings of a previously healthy girl who died suddenly at the age of 9 weeks. Severe cardiac enlargement was observed on post-mortem MRI (Fig. 1a) and at autopsy. Cardiomyocyte disarray was observed with distinct (peri)nuclear changes consistent with hypertrophic cardiomyopathy (HCM), by light microscopy (Fig. 1b). No signs of mitochondrial or storage diseases were seen by electron microscopy (not shown). There was no history of maternal diabetes mellitus and family history was negative for sudden death, cardiomyopathy or heart failure. DNA testing revealed compound heterozygous truncating *MYBPC3* mutations: c.2373dup p.(Trp792Valfs*41), inherited from the mother, and c.2827C > T p.(Arg943*), inherited from the father. Both are Dutch founder mutations and are, in a heterozygous state, the most common causes of (adult onset) HCM in the Netherlands [1]. Identification of these pathogenic mutations not only explained the neonatal onset of HCM in the index, but also allowed for the screening of other relatives at risk for (adult onset) HCM. Fatal neonatal HCM caused by homozygous or compound heterozygous pathogenic mutations in one of the sarcomeric protein encoding genes is thought to be rare, but the exact prevalence is unknown [2–5]. Although other causes of neonatal cardiac hypertrophy must be considered (such as maternal diabetes, RASopathies, mitochondrial or storage disorders), we believe this case underlines the value of testing for genetic causes of HCM.

**Fig. 1:**
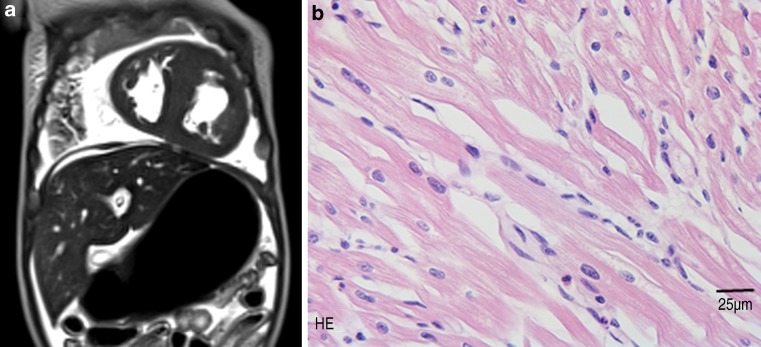
**a** Post-mortem MRI showing severe cardiac enlargement. **b** Light microscopy displaying cardiomyocyte disarray with distinct (peri)nuclear changes consistent with hypertrophic cardiomyopathy. *HE* Haematoxylin and eosin stain

## References

[1] Christiaans I, Nannenberg EA, Dooijes D (2010). Founder mutations in hypertrophic cardiomyopathy patients in the Netherlands. Neth Heart J.

[2] Wessels MW, Herkert JC, Frohn-Mulder IM (2015). Compound heterozygous or homozygous truncating MYBPC3 mutations cause lethal cardiomyopathy with features of noncompaction and septal defects. Eur J Hum Genet.

[3] Xin B, Puffenberger E, Tumbush J (2007). Homozygosity for a novel splice site mutation in het cardiac myosin-binding protein C gene causes severe neonatal hypertrophic cardiomyopathy. Am J Med Genet A.

[4] Zahka K, Kalidas K, Simpson MA (2008). Homozygous mutation of MYBPC3 associated with severe infantile hypertrophic cardiomyopathy at high frequency among the Amish. Heart.

[5] Lekanne Deprez RH, Muurling-Vlietman JJ, Hruda J (2006). Two cases of severe neonatal hypertrophic cardiomyopathy caused by compound heterozygous mutations in the MYBPC3 gene. J Med Genet.

